# Mammalian Tribbles Homologs at the Crossroads of Endoplasmic Reticulum Stress and Mammalian Target of Rapamycin Pathways

**DOI:** 10.1155/2013/750871

**Published:** 2013-12-30

**Authors:** Robyn Cunard

**Affiliations:** ^1^Research Service and Division of Nephrology-Hypertension, Veterans Affairs San Diego Healthcare System, Veterans Medical Research Foundation, Mail Code 151, 3350 La Jolla Village Drive, San Diego, CA 92161, USA; ^2^Department of Medicine, University of California San Diego, La Jolla, CA 92093, USA

## Abstract

In 2000, investigators discovered Tribbles, a Drosophila protein that coordinates morphogenesis by inhibiting mitosis. Further work has delineated Xenopus (Xtrb2), Nematode (Nipi-3), and mammalian homologs of Drosophila tribbles, which include TRB1, TRB2, and TRB3. The sequences of tribbles homologs are highly conserved, and despite their protein kinase structure, to date they have not been shown to have kinase activity. TRB family members play a role in the differentiation of macrophages, lymphocytes, muscle cells, adipocytes, and osteoblasts. TRB isoforms also coordinate a number of critical cellular processes including glucose and lipid metabolism, inflammation, cellular stress, survival, apoptosis, and tumorigenesis. TRB family members modulate multiple complex signaling networks including mitogen activated protein kinase cascades, protein kinase B/AKT signaling, mammalian target of rapamycin, and inflammatory pathways. The following review will discuss metazoan homologs of Drosophila tribbles, their structure, expression patterns, and functions. In particular, we will focus on TRB3 function in the kidney in podocytes. This review will also discuss the key signaling pathways with which tribbles proteins interact and provide a rationale for developing novel therapeutics that exploit these interactions to provide better treatment options for both acute and chronic kidney disease.

## 1. Introduction

In the age of personalized and targeted medical therapies [1], the treatment of both acute and chronic kidney disease (CKD) remains a formidable challenge. Our treatment options in these diseases are limited and often rely on basic immunosuppression with corticosteroids, steroid-sparing medications, and supportive measures. Both acute kidney injury [2–4] and chronic kidney disease [5–9] are associated with the activation of multiple signaling pathways, which can contribute to persistent organ injury. It is likely that therapeutics that regulate these signaling cascades can be developed to provide more effective and specific approaches for treating diverse kidney diseases. Work over the last decade has elucidated a number of novel signaling molecules that dampen the activation of potentially harmful signaling cascades. One such protein, tribbles may function at the intersection of multiple stress-activated pathways including the mammalian target of rapamycin (mTOR), endoplasmic reticulum (ER) stress, and macro-autophagy pathways. In the following review, we will first discuss metazoan homologs of Drosophila tribbles, their structure, expression patterns, and functions. We will also review the key signaling pathways with which tribbles proteins interact and provide a rationale for developing novel therapeutics that exploit these interactions to provide better treatment options for both acute and chronic kidney disease.

## 2. TRB Family Members

Tribbles was first described in 2000 as a Drosophila (fruit fly) protein that coordinates morphogenesis by inhibiting mitosis [10–12]. The moniker was based on observations that tribbles mutants (knockdown of tribbles) enter mitosis early, and have over-proliferation of mesodermal cells, and resemble the highly proliferating Tribbles organisms encountered on the science fiction Star Trek television show [10, 13]. Tribbles arrests cells in the G2 phase of the cell cycle, by binding to and inducing proteasome-mediated degradation of *String and Twine*, cyclin-dependent kinase 25 (CDC25) phosphatases [11]. In the ovary, Tribbles targets migration of the slow border cells (*Slbo*), (CAAT enhancer binding protein, C/EBP homolog), for rapid degradation via the ubiquitin-proteasome pathway [14, 15]. These early fly studies demonstrated that Tribbles was important in synchronizing sequential cytoskeletal arrangements necessary for coordinating cell division, fate, and morphogenesis [16, 17]. Follow up work in Drosophila has demonstrated that tribbles plays a role in fly memory, central nervous system (CNS) development, and bristle formation (bundles of actin filaments) [18, 19]. Tribbles also reduces Notch signaling during bristle patterning [17].

In Xenopus (African clawed frog), investigators discovered a tribbles homolog Xtrb2, which plays a significant role in development. They discovered two alternatively spliced cDNA sequences, a short and long Xtrb2, which are differentially expressed. Surprisingly, depletion of Xtrb2 delays cell division and induces abnormalities in somite and eye development. These studies suggested that Xtrb2 plays a role in the progression of mitosis and proper formation of the nervous system [20]. Interestingly, GFP-tagged Xtrb2 was transiently associated with mitotic spindles during cell division [20]. In Caenorhabditis elegans (nematode), investigators identified nipi-3, a tribbles-like protein. Nipi-3 is upregulated in response to fungal infection and may play a role in the innate immune response [21]. Follow-up studies have suggested that nipi-3 functions upstream of a glucose regulated protein 78/binding immunoglobulin protein (GRP78/BiP) homolog to induce expression of antimicrobial peptides in a mitogen-activated protein kinase (MAPK)-dependent manner [22, 23]. Thus, these early studies in primitive metazoans demonstrated that tribbles both promotes and blocks cell division, is involved in cytoskeletal dynamics, interacts with the ubiquitin-proteasome degradation system, and regulates innate immunity. The diversity of function is quite remarkable.

In mammalian cells, prior to the discovery of tribbles, investigators identified proteins that were subsequently shown to be homologs of Drosophila tribbles. In 1997, Wilkin and colleagues described c5fw (clone 5 Françoise Wilkin) and c8fw, proteins upregulated in dog thyroid cells chronically treated with thyroid stimulating hormone [24, 25]. In neuronal cells, a homologous protein, novel kinase-like gene induced during cell death (NIPK), increased in neurons deprived of neuronal growth factor and treated with the calcium ionophore, A23187 [26]. The same protein, p65 interacting inhibitor of nuclear factor kappa light chain-enhancer of activated B cells (NF-*κ*B, SINK), was shown to inhibit p65 phosphorylation by Protein Kinase A (PKA, cAMP-dependent kinase) and sensitize cells to tumor necrosis factor (TNF)-induced apoptosis [27]. SKIP3, another homolog, was discovered in human lung, colon, esophageal, and breast tumors [28]. Further work has delineated three mammalian homologs of Drosophila tribbles, which include TRB1 (Trib1, c8fw, SKIP1), TRB2 (Trib2, c5fw, SKIP2), and TRB3 (Trib3, NIPK, SINK, or SKIP3) [24, 26–29]. Notably, human and mouse TRBs share significant amino acid homology and the high evolutionarily conserved sequences support their importance as critical regulators of cellular processes. Among the human TRB proteins, TRB1 and TRB2 share 71% homology, TRB1 and TRB3, 53%, and TRB2 and TRB3 share 54% homology [30].

## 3. Structure

**Figure 1:**
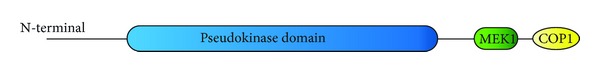
The basic protein structure of TRB isoforms. There is significant sequence divergence in the N-terminal domain among tribbles homologs. The pseudokinase domain has what is believed to be a kinase-dead catalytic loop [26], and the C-terminal domain has MEK1 (binds MAPKK, [44]) and COP1 (binds ubiquitin ligases) binding motifs. The pseudokinase domain is important for protein-protein interactions between transcription factors.

Early work on TRBs demonstrated that their sequences were similar to classic serine-threonine protein kinases [24, 26, 31]. TRBs have an N-terminal domain, central kinase-like domain, and C-terminal protein-binding domain (Figure 1). However, investigators discovered significant variations in the amino acids in the ATP binding pocket and the kinase catalytic core (review in [18, 30, 32]). Early studies suggested that TRBs could bind to kinase-dependent proteins, but they lacked kinase activity [24, 26]. Thus, TRBs are classified as pseudokinases, and belong to a family of proteins which include Integrin-linked kinase (ILK), Janus tyrosine kinases (JAKs), ErbB3/HER3, and Erythropoietin-producing hepatocyte kinases (EphB6) [33]. The mechanisms of action of TRB homologs remain incompletely understood, though investigators have hypothesized that tribbles homologs function as docking kinases [34], scaffolds that balance complex signaling pathways [35], or allosteric activators of protein kinases [36]. Interestingly, the WNK (with no (K) Lys) proteins lack conserved catalytic lysine residues required for ATP binding [37]. Despite these variations in the catalytic domains, WNK proteins are active kinases and play a significant role in kidney ion transport and blood pressure control [38]. CASK and ErbB3/HER3 were also originally described as pseudokinases, and later discovered to possess kinase activity [39, 40]. It is likely that studies investigating TRBs' three-dimensional structures will be necessary to determine potential targets and demonstrate kinase activity [41].

There is significant sequence divergence in the N-terminal domains of TRB family members, but the C-terminal domains have two conserved sequences: an E3 ubiquitin ligase constitutive photomorphogenic protein 1 (COP1) site, which binds an E3 ubiquitin ligase, (also known as RING finger and WD repeat domain protein 2, RFWD2) [42, 43], and a MEK1 (Mitogen activated protein kinase kinase, MAPKK) binding site which mediates interactions with multiple MAPKKs including mixed lineage kinase 3 (MLK3) [44, 45]. TRB3 has a D-box destruction motif, which is important for ubiquitin ligase anaphase-promoting complex/cyclosome APC/C^CDC20^ and APC/C^Cdh1^-dependent proteolysis [46]. Additionally, Imajo and Nishida have shown that TRB1 interacts with retinoic acid receptor-*α* (RAR*α*) and retinoid X receptor-*α* (RXR*α*) heterodimers through its pseudokinase domain [47].

## 4. Expression Patterns

TRB1 is expressed in the liver, kidney, heart, brain, skin, small intestine, bone marrow (BM), peripheral blood leukocytes (PBL), especially monocytes, macrophages and B cells, thyroid gland, white adipose tissue (WAT), and pancreas [48–51]. TRB2 is expressed in the thymus, heart, brain, kidney, lung, skin, spleen, PBL (T and B lymphocytes), and WAT [48, 49]. TRB3 is most highly expressed in the liver and hematopoietic compartments, such as BM, PBL (B cells), spleen, thymus, prostate, heart, kidney, lung, skin, small intestine, WAT, neurons, skeletal muscle, and stomach [26–28, 48, 52–54]. When detailed studies using sensitive means of detection (i.e., real-time PCR) are used, the mRNA of TRB isoforms is ubiquitously expressed. In developmental studies, investigators have observed the expression of TRB2 in a variety of organs during gestation, including the kidneys, mesonephros, testes, heart, eyes, thymus, blood vessels, muscle, bones, tongue, spinal cord, and ganglions [55, 56]. TRB1 was less abundantly expressed in embryonic and adult kidneys than TRB2, and TRB3 was undetectable in embryonic kidneys [55]. Indeed, TRB isoform expression is highly regulated and very context-dependent. TRBs function in both the cytoplasm and nucleus, though investigators have recognized that TRB1 and TRB3 preferentially localize to the nucleus, whereas TRB2 is more often detected in the cytoplasm [32].

TRB3 expression is generally regulated at the transcriptional level [27, 52, 57]. Interestingly, in mouse embryonic stem cells, TRB1 belongs to a subset of mRNA species with extremely short half lives (<1 hr), the authors suggested that the mRNA's half-life was related to its physiological role, that is, the shorter the half-life, the more critical its role [58]. Studies also suggest that the TRB3 protein is short-lived and protein levels are regulated by proteasome-dependent degradation by the E3 ubiquitin ligase seven in absentia homolog 1 (SIAH1) [59], ubiquitin ligase anaphase-promoting complex/cyclosome Cdh1 (APC/C^cdh1^) [46], and cysteine-dependent aspartate-directed protease 3 (caspase 3) [60].

## 5. Functions of TRB Isoforms

As previously discussed, tribbles homologs have not yet been shown to have specific kinase function. However, TRB family members play a role in the differentiation of macrophages [51], lymphocytes [52], muscle cells [61], adipocytes [62–64], and osteoblasts [65]. TRBs also coordinate a number of critical cellular processes including glucose and lipid metabolism, inflammation, cellular stress, survival, apoptosis, and tumorigenesis. Moreover, they regulate and interact with a number of transcription factors.  Table 1 describes these transcription factors which include: activating transcription factor 4 (ATF4) [66–68], ATF5, C/EBP*β* [63, 69], C/EBP*α* [70, 71], C/EBP homologous protein (CHOP) [57], NF-*κ*B [27, 50] Forkhead box protein/forkhead in rhabdomyosarcoma O1 (FOXO1) [72], FOXO3a [73], FOXP3 [74], PPAR*γ* [75], RAR*α*, and RXR*α* [47]. However, the function of TRB isoforms in the kidney is not completely understood.

### 5.1. TRB1

The only investigation of TRB1 and its association with renal disease demonstrated that it is a peripheral blood biomarker of chronic immune-mediated rejection in kidney transplant patients [49]. The investigators also observed higher TRB1 expression in renal biopsies with rejection and in a rodent model of chronic cardiac vasculopathy, suggesting that it could be a useful biomarker for other solid-organ transplants. Notably, in their study, TRB1 was primarily expressed by antigen-presenting cells and activated endothelial cells [49]. The transcription factor FOXP3 is a specific marker of regulatory T cells (Tregs) and its deficiency is associated with autoimmune diseases and inflammation [76]. FOXP3^+^ Tregs may facilitate graft tolerance and promote long-term organ transplant survival [77]. Recent work suggests that TRB1 binds to Foxp3 in Tregs [74]; however, the significance of this interaction has not yet been fully explored. TRB1 expression increases in chronically inflamed human atherosclerotic arteries and reduces vascular smooth muscle cell proliferation and chemotaxis [35, 78]. TRB1 also regulates lipoprotein metabolism [79]. Hepatic-specific overexpression of TRB1 reduces plasma triglycerides (TG) and cholesterol by reducing very low-density lipoprotein (VLDL) production, and TRB1 knockout mice have elevated TG and cholesterol [79]. Not surprisingly, variations in TRB1 loci in humans are associated with increased plasma lipoproteins and risk of coronary artery disease [51, 80, 81]. In white adipose tissue (WAT) TRB1 expression is upregulated during acute (lipopolysaccharide, LPS) and chronic inflammation (*db/db* mice), and TRB1 heterozygous knockout mice have impaired cytokine gene expression in white adipose tissue (WAT) and are protected from weight gain and adiposity when fed a high fat diet. The investigators further demonstrated that TRB1 interacts with the NF-*κ*B subunit RelA (p65) and affects RelA transcriptional activity via direct physical interaction upon promoter recruitment. Thus, in adipocytes, TRB1 is a nuclear transcriptional coactivator for NF-*κ*B, and induces the expression of pro-inflammatory cytokines [50]. These studies suggest that in the liver, TRB1 positively impacts lipoprotein metabolism, but in WAT TRB1 increases inflammatory cytokine expression, which could ultimately contribute to organ dysfunction.

A number of studies have confirmed that macrophages can be differentially activated into different functional subtypes. M1 cells are classically activated macrophages with pro-inflammatory functions. In contrast, M2 macrophages are alternatively activated, exert anti-inflammatory effects, and are involved in wound healing, tissue repair, and cancer cell growth [82–84]. Early work suggested that in macrophages, TRB1 negatively regulates C/EBP*β* (nuclear factor of IL-6, NF-IL6) expression and LPS-stimulated TRB1-deficient macrophages have higher expression of prostaglandin E synthase and Lipocalin 2/Neutrophil gelatinase-associated lipocalin (Lcn2/Ngal) and lower IL-12 expression [85]. TRB1 may also play a role in macrophage migration [86]. Recent follow up studies demonstrate that TRB1 plays a critical role in differentiation of tissue-resident M2-like macrophages and eosinophils by regulating C/EBP*α* expression [51]. Dr. Akira's laboratory demonstrated that TRB1 knockout mice have less adipose tissue and this is associated with augmented lipolysis. Interestingly, this defect was rescued by supplementation of M2 macrophages, demonstrating that TRB1 and M2 macrophages play a critical role in adipose tissue maintenance and suppression of metabolic disorders. TRB1 may also inhibit host responses to entamoeba infections [87]. These studies all support the concept that TRB1 plays a central role in the cross-talk between adipose tissue, the immune system, and metabolic homeostasis [88, 89].

### 5.2. TRB2

In the fetal kidney, TRB2 is expressed in the comma, S-shaped bodies [90], podocytes, and the mesangium of the developing glomeruli, as well as in the ureteric bud tips. However, TRB2 mutant mice are functionally and structurally normal. The authors suggested that TRB2 may play a minimal role during kidney and mouse development [55]; however, the possibility of redundant TRB function was not completely explored, nor were the mice stressed. In murine hematopoietic stem cells, retroviral expression of TRB2 induces acute myelogenous leukemia and degradation of C/EBP*α* [70, 71]. TRB2 maintains the oncogenic properties of melanoma cells, and TRB2 knockdown reduces cell proliferation, colony formation, and wound healing [73]. In contrast, in certain cytokine-dependent hematopoietic cell lines, TRB2 modulates apoptosis, but this effect is absent in adherent cells, which is possibly related to survival signals associated with adhesion [91]. TRB2 suppresses adipocyte differentiation by inhibiting AKT and C/EBP*β* [69]. Downregulation of TRB2 potentiates LPS-induced IL-8 production via MAPK pathways [92], and TRB2 expression is highly upregulated in human atherosclerotic plaques. In primary human monocyte-derived macrophages, TRB2 reduces IL-10 mRNA expression, suggesting that TRB2 may play a role in plaque instability [93]. Thus, the functions of TRB2 are quite diverse, but clearly TRB2 plays a role in inflammation and cellular differentiation including tumorigenesis.

### 5.3. TRB3

To date, TRB3 has been the most intensely studied mammalian TRB isoform. TRB3 is expressed in the kidney [28, 48], and our group has observed TRB3 expression in podocytes and tubular cells [53]. In the kidney, our studies suggest that TRB3 inhibits inflammatory cytokines and chemokines, as TRB3 inhibits podocyte expression of monocyte chemokine protein 1 (MCP-1) [53]. Kuo and colleagues have similar findings, and knockdown of TRB3 in sensitized mast cells increases expression of IL-6, MCP-1, TNF*α*, and IL-4, suggesting again that TRB3 may negatively regulate the expression of pro-inflammatory cytokines and chemokines [94]. Early work by Marc Montminy's group demonstrated that TRB3 binds to and masks phosphorylation of Protein Kinase B/AKT at Threonine (Thr)^308^ and Serine (Ser)^473^ residues, thereby reducing insulin-stimulated glucose output in liver cells [95]. Later in this review, we will discuss the impact of AKT phosphorylation on kidney pathophysiology. *In vivo*, TRB3 expression increases in the livers of fasted mice and functions to increase glucose output [95, 96], though there has been controversy regarding the ability of TRB3 to block phosphorylation of AKT [75, 97]. TRB3 also inhibits insulin-induced activation of S6 kinase 1 by mammalian target of rapamycin [98], and stimulates liver lipolysis by promoting the degradation of acetyl-Coenzyme A carboxylase (ACC), the rate-limiting enzyme of fatty acid synthesis [42]. TRB3 is upregulated in the skeletal muscle of patients with Type II diabetes and TRB3 over-expression in muscle cells blocks insulin-stimulated glucose transport and impairs phosphorylation of AKT, extracellular-signal regulated kinase (ERK), and insulin receptor substrate-1 (IRS1) [54]. TRB3 impacts glucose uptake and oxidation oppositely in muscle and fat, and Liu and colleagues have postulated that TRB3 may function as a sensor of nutrient availability [99]. Interestingly, recent work suggests that TRB3 inhibition may improve insulin sensitivity *in vivo* primarily in a PPAR*γ*-dependent manner, independent of changes in AKT activity [75]. These studies demonstrate that TRB3 regulates insulin sensitivity and lipolysis, and may be an excellent therapeutic target for insulin resistance [42, 95, 98, 100–102].

## 6. TRB Knockout Mice

All three TRB isoforms have been successfully knocked-out in mice and investigators have postulated that the viability of each knockout, could be related to compensation by other TRB family members. Though to date, upregulation of other TRB isoforms in the single TRB2 and TRB3 knockout mice has not been observed [48, 55]. The development of double and triple knockout mice should reveal whether TRB isoforms have redundant function [30, 55]. In 2007, Okamoto et al. [48] developed a strain of TRB3 knockout mice, and they did not observe alterations in fasting and postprandial glucoses, lipids, insulin, leptin, hepatic insulin sensitivity or lipid metabolism. They proposed that constitutive loss of TRB3 was not sufficient to alter the maintenance of glucose and energy homeostasis, and future challenge studies were necessary to evaluate TRB3 function [48]. Dr. Shizuo Akira's group has developed constitutive knockout of TRB1, TRB2, and TRB3 mice [51] and each of these mice strains are viable. Though there are reports of higher perinatal mortality in TRB1 homozygous knockout mice on the C57BL/6 background [50], and the female TRB1 knockout mice are infertile [85]. As discussed, many studies have suggested that TRB3 can induce insulin-resistance; thus, it will be of interest in the future to evaluate whether constitutive TRB3 knockout alters insulin signaling and fasting glucoses in murine models of Type 1 and 2 diabetes. Örd and colleagues independently developed another strain of TRB3 knockout mice and discovered that TRB3 regulates mast cell survival and function [103]. TRB2 knockout mice also develop normally [55]; however, investigations of these mice in acute and chronic disease models have not yet been published.

Recently, two groups have shown that partial TRB3 gene silencing (by RNAi technology) alleviates diabetic cardiomyopathy and diabetic atherosclerosis. In a model of diabetic atherosclerosis, male apoE/low density lipoprotein (LDL) receptor double knockout mice were fed a high fat diet and then treated with low dose STZ. TRB3 knockdown reduced the extent of atherosclerosis, increased plaque stability, and reduced macrophage apoptosis and migration [104]. In a model of diabetic cardiomyopathy, rats were treated with a high fat diet and STZ. The rats with TRB3 knockdown had improved insulin resistance and cardiac function [105]. In both studies, the efficiency of TRB3 knockout in the aorta and heart was about 70%, and serum glucoses were dramatically improved. Thus, in these studies, improvements in diabetic cardiomyopathy and atherosclerosis could have been related to improved metabolic control. In murine heart tissue, TRB3 expression also increases in experimental myocardial infarction. Interestingly, transgenic mice with cardiac-specific overexpression of TRB3 had reduced cardiac glucose oxidation rates and were sensitized to infarct expansion and cardiac myocyte apoptosis in the infarct border zone after myocardial infarction [106]. TRB3 appears to have both beneficial and deleterious effects in multiple tissues. Our studies in lymphocytes and the kidney suggest that TRB3 inhibits inflammation, and we postulate that augmented expression of TRB3 may improve outcomes in acute and chronic kidney diseases. Further studies with TRB3-specific over-expression in the kidney, especially in podocytes and tubular cells will be of great interest to identify whether manipulation of TRB3 expression is therapeutically efficacious in both acute and chronic kidney diseases.

## 7. TRB3 and Cellular Survival and Apoptosis

Early studies suggested that TRB3 induces apoptosis. In brain tumor cells, TRB3 is upregulated by cannabinoids and activates autophagy and caspase-mediated apoptotic pathways [107]. Interestingly, this effect was limited to transformed cells and not observed in nontransformed neuronal cells [107]. TRB3 also mediates human monocyte-derived macrophage apoptosis [108], and knockdown of TRB3 reduces endoplasmic reticulum (ER) stress-induced apoptosis [57]. TRB3 promotes apoptosis in pancreatic *β*-cells [45] and chondrocytes [109]. However, in podocytes [53] of the kidney, we have not observed apoptosis when TRB3 is over-expressed. In fact, TRB3 does not universally cause cell death. TRB3 is induced five-fold by erythropoietin and is associated with erythroblast survival [62]. In postmitotic neuronally differentiated PC12 cells, coexpression of TRB3 with ATF4 prevents ATF4-induced apoptosis [110] and TRB3 can function as a pro-survival factor in glucose-starved PC-3 prostate cancer cells [111] and bone marrow-derived mast cells [103]. Thus, TRB3's effects on survival or apoptosis are likely cell type and context dependent. Indeed, Shimizu and colleagues postulate that TRB3 serves as a switch between cellular survival and apoptosis. Caspases play a central role in the execution-phase of apoptosis, and they demonstrated that TRB3 prevents the activation of caspase 3 by nuclear translocation of procaspase 3. However, in conditions of prolonged stress, TRB3 is cleaved by caspase 3 and no longer inhibits caspase 3-induced apoptosis [60]. Again, TRB3 function is very context and cell type-dependent, suggesting that TRB family members may sense, integrate, and respond to diverse signals to promote homeostatic function.

## 8. Cell Cycle

During the development and differentiation of multicellular organisms, precise control over the timing of cell cycle progression is critical, as premature cellular divisions can disrupt morphogenesis [112]. Therefore, cells have developed a number of mechanisms to prevent premature entry into the synthesis (S) and mitosis (M) phases of the cell cycle [113]. The initiation of mitosis is controlled by a catalytic subunit cyclin-dependent kinase 2 (CDC2), and a regulatory subunit, Cyclin B. CDC25C dephosphorylates Cyclin B-CDC2 and triggers entry into mitosis. Early work suggested that Drosophila tribbles slows cell cycle progression by inducing the degradation of a CDC25C homolog [10, 11] and Tribbles overexpression in the Drosophila wing greatly reduces the number of cells per wing [11]. In mammalian cells, TRB3 deletion upregulates the expression levels of CDC20 and CDC25A [46], and TRB3 regulates the stability of CDC25A, a cell-cycle regulator that is degraded in response to DNA damage [114, 115]. However, it is not clear whether TRB3-induced degradation of CDC25A affects cell cycle progression. In lymphocytes, we have shown that TRB3 blocks cells in the G2 phase of the cell cycle [52], and transcriptional studies suggest that TRB3 inhibits activation of the Cyclin B1 promoter [116]. In a similar manner, in endothelial cells TRB3 mediates homocysteine-induced inhibition of cell cycle progression by up-regulating expression of p27(kip1) [117]. Cell cycle progression requires multiple levels of regulation and it is likely that TRB3 modulates the expression of other key cell cycle modulators including p21 and p53.

## 9. Inflammation

Early investigations by Kiss-Toth's group demonstrated that TRBs interact with MAPK kinases to regulate MAPK [35]. They demonstrated that all TRB isoforms regulate inflammatory signaling networks, by binding to c-Jun N-terminal kinases (JNKs), and p38 to regulate IL-8 production [118]. As previously discussed, TRB1 is a novel binding partner of FOXP3, a master regulator of regulatory T cells [74]; thus, it is likely that TRB1 plays a significant role in immune cell function. Furthermore, TRB1 deficiency impairs cytokine gene expression in white adipocytes [50], and TRB1 is involved in cytokine and chemokine expression of mast cells [94] and polarization of M2 macrophages [51]. *Helicobacter pylori* is a bacterial pathogen that causes stomach inflammation and in gastric epithelial cells TRB3 enhances Toll-like receptor 2 (TLR2)-mediated NF-*κ*B activation and chemokine induction in response to *H. pylori* LPS [119]. Our studies have also demonstrated that TRB3 mediates anti-inflammatory effects in lymphocytes, podocytes, and renal tubular cells [52, 53, 116]. These studies all support the concept that TRBs play a significant role in immunity. Similar to their effects on insulin sensitivity and metabolism, their seemingly contradictory effects in diverse cell types likely function to control the extent of the inflammatory response and may provide a means of balancing cross-talk between solid organs and the immune system.

## 10. Regulation of Expression of TRB3

Multiple cellular stressors including nutrient and essential amino acid deprivation, activation of phosphatidylinositol-3-kinase (PI3K), ER stress, thapsigargin, free fatty acids, fasting, cadmium, TNF, phorbol esters, arsenite, nerve growth factor depletion, hypoxia, insulin, oxidized LDL, ethanol, and IL-3 augment TRB3 expression [26–28, 53, 57, 66, 67, 72, 95, 103, 108, 111, 120–123]. In contrast, phosphodiesterase inhibition, dexamethasone, cyclic adenosine monophosphate (cAMP), IL-3 deprivation, and activation of FOXO-1 reduce TRB3 expression [63, 72, 103]. Interestingly, in prostate cancer cells with constitutively active PI3K signaling, TRB3 expression is down-regulated in response to rapamycin (an mTOR complex 1, mTORC1 inhibitor) [111]. In neuronal cells, chronic lithium treatment downregulates TRB3 expression suggesting that TRB3 could play a role in bipolar affective disorder [124]. Although, TRB3 has not yet been shown to directly regulate p53 activity, in human colon cancer cells, genotoxic stress (chemotherapeutic agents) down-regulates TRB3 in a p53-dependent and p53-independent manner [125]. Acetylation of TRB3 may also regulate its activity. Yao and colleagues demonstrated that TRB3 was upregulated and hypoacetylated in a rat model of prenatal alcohol exposure [126]. Thus, the regulation of TRB3 and its other family members is extremely complex, again related to its function as a regulator and integrator of many cellular signaling pathways.

Investigators have characterized 3 tandem 33 base pair (bp) repeats in the human TRB3 promoter [57, 67]. Each of these repeats have a C/EBP-ATF composite site that resembles sequences in the Nutrient Sensing Response Element (NSRE-1) in the asparagine synthetase promoter and the Amino Acid Response Element (AARE) in the CHOP promoter [57, 67]. Cellular stress upregulates CHOP and ATF4, which in turn activate the 33 bp repeats in the human TRB3 promoter [57, 67]. In our studies in lymphocytes and podocytes, both C/EBP*β* and CHOP bind to the proximal TRB3 promoter and drive TRB3 transcription [52, 53]. Other investigations suggest that PPAR*α* [96] and FOXO1 also regulate TRB3 promoter activity [72].

## 11. Mechanism of Action

Before we discuss the effects of TRB3 on AKT phosphorylation and function, it is important to provide a framework for understanding AKT, as it plays a central role in transducing signals that affect cell survival, proliferation, inflammation, and metabolism. Moreover, AKT regulates hundreds of downstream targets (review in [127]), many with opposing functions. AKT is a serine-threonine kinase and full activation of AKT by growth factors, including insulin requires phosphorylation of Ser^473^ residues by the mTOR complex 2 (mTORC2) and phosphorylation of Thr^308^ residues by 3-phosphoinositide-dependent protein kinase-1 (PDK1) [128]. Phosphorylation of AKT at Thr^450^ residues may also impact kinase activity and stability [129].

AKT is activated in the cortices of diabetic mice by multiple stimulators including Ang II and reactive oxygen species (ROS) [130–134], and stimulates the formation of extracellular matrix, including fibronectin [135]. Early work suggested that lower AKT phosphorylation reduces cellular survival [136, 137]; however, more recent work suggests that differential phosphorylation of AKT at Thr^308^ and Ser^473^ residues likely exerts diverse effects. Indeed, rat glomeruli injured with protamine sulfate have higher albumin permeability associated with increased phosphorylation of AKT^Ser473^ and blockade of mTORC2 reduces phosphorylation of AKT^Ser473^ and albumin release [138]. In parallel, high glucose conditions and advanced glycation endproducts increase phosphorylation of AKT^Ser473^ in rat podocytes and this is associated with cleaved caspase 3 and apoptosis [139]. Inflammatory mediators such as TNF*α* and IL-6 increase phosphorylation of AKT and reduce nephrin promoter activity in reporter murine podocytes [140]. Thus, the relevance of differential phosphorylation of AKT on Thr^308^, Ser^473^, and Thr^450^ residues is complex and not completely understood. Recent studies suggest that downstream substrates may require different levels of AKT activity, and differential AKT phosphorylation patterns may provide a mechanism for integrating diverse cellular stimuli. Moreover, differential phosphorylation of AKT at Thr^308^ and Ser^473^ residues may confer substrate specificity and facilitate splitting of upstream signals into discrete outputs [141, 142].

At this time, despite the lack of evidence of kinase activity, investigators have elucidated a number of functions of TRB family members, most of which involve direct protein/protein interactions and degradation of target molecules. As previously discussed, TRB3 binds to and masks phosphorylation of AKT at Thr^308^ and Ser^473^ residues. This work has been confirmed by a number of groups [61, 95, 102, 123, 143, 144] and disputed by others [75, 97]. Indeed in lymphocytes, we have not observed inhibition of phosphorylation of AKT at Thr^308^ residues associated with over-expression of TRB3 [116]. Our recent work in podocytes, tubular cells, and a murine model of Type 1 diabetes suggests that TRB3 selectively inhibits phosphorylation of AKT at Ser^473^ residues [145], and other groups have similar observations [146]. These findings suggest that TRB3 may fine-tune AKT activation [143] to modulate multiple physiological inputs. To add a layer of complexity, FOXO1 an AKT-target protein, also plays an important role in hepatic glucose output [147]. Unphosphorylated FOXO1 localizes in the nucleus and drives the transcription of genes important in gluconeogenesis and glycogenolysis. Through AKT, insulin induces the phosphorylation of FOXO1, promoting its exclusion from the nucleus, ubiquitination, and degradation [148–150]. Accili's group demonstrated that FoxO1 activates AKT and inhibits TRB3 expression in hepatocytes [72]. Thus, there is significant cross-regulation among insulin, AKT, FOXO1, and TRB3. We hypothesize that the complex regulation of TRB3 and its interaction with multiple metabolic signaling cascades may provide a mechanism of harmonizing nutrient availability with complex physiologic programs such as growth, proliferation, and differentiation [143].

TRBs do not have a DNA-binding domain, but they interact with transcription factors, especially members of the basic region leucine zipper (bZIP) family and can inhibit their action by direct binding or by inducing proteolysis. TRB1 and TRB2 induce degradation of C/EBP*α*, but TRB3 doesn't [43, 70, 71, 151]. TRB2 and TRB3 bind to C/EBP*β* [63, 69] and TRB3 binds CHOP [57] and ATF4 and regulates ATF4's activity [28, 66, 67]. TRB family members also bind factors that regulate NF-*κ*B and AP-1 [27, 35, 152], and TRB isoforms bind nuclear receptors including PPAR*γ*, RAR*α*, and RXR*α* [47, 64].

Besides binding to and/or mediating the proteolysis of key transcription factors TRB3 also degrades ACC1 [42], Smad ubiquitin regulatory factors (SMURFs) [65, 153], and caspase-3 [60]. TRB1 and 3 bind to MAPKK and regulate their activity [35]. In vascular smooth muscle cells, TRB1 binds to MKK4 and inhibits JNK activity [35, 78]. TRB3 also interacts with the mixed lineage kinase 3 (MLK3) and compromises mitochondrial integrity and suppresses cellular survival [45]. TRB3 binds to the Bone Morphogenic Proteins (BMP) Type II (BMPRII) receptor and promotes BMP4 signaling [65]. The general theme of these studies is that TRB family members alter transcriptional and posttranscriptional events by interacting with transcriptional and signaling regulators and either block their phosphorylation or target the proteins for degradation.

## 12. TRB3 and Cancer

Early work demonstrated that TRB3 (SKIP3) was expressed in human lung, colon, esophageal, and breast tumors [28, 154]. The Notch signaling network is an evolutionarily conserved intercellular signaling pathway that plays a role in kidney development and disease [155]. Recent investigations have demonstrated that TRB3 is a master regulator of Notch through the MAPK-ERK and Transforming Growth Factor *β* (TGF*β*) pathways, and is required for the growth of basal-like breast cancer [156, 157]. Indeed, TRB3 may serve as a prognostic marker in breast cancer [158–161]. In HepG2 cells, TRB3 plays a role in tumor progression and metastases, by augmenting TGF*β*-SMAD3-transcriptional activity and inducing epithelial-mesenchymal transition (EMT) [65, 153]. TRB3 expression is upregulated in nonsmall cell lung cancer (NSCLC) and expression correlates with poor survival in patients [157]. In hepatocellular carcinoma cells and gliomas, cannabinoids increase TRB3 expression [107, 162]. In human hepatoma cells, a novel NF-*κ*B inhibitor, dehydroxymethyl-epoxyquinomicin (DHMEQ) promotes TRB3 mRNA induction and inhibits cell growth and apoptosis [163]. Investigators have postulated that TRB3 may facilitate the growth of cells in nutrient-limiting conditions [28, 111]. TRB3 may also play a role in cell cycle control, cell survival, DNA repair, and genome stability by interacting with Ct interacting protein (CtIP) [154] and polydeoxynucleotide cytidine deaminases APOBEC3A and APOBEC3C. These interactions inhibit nuclear DNA editing, suggesting that TRB3 may be an important guardian of genome integrity [164].

TRB1 is overexpressed in acute myelogenous leukemia (AML) [165], can induce AML in mice [71, 166], and a TRB1 somatic mutation was observed in a human case of Down syndrome-related acute megakaryocytic leukemia [167]. In contrast, TRB2 expression is upregulated in only a subset of patients with AML [70, 168] and those with T acute lymphoblastic leukemia [169]. TRB2 may also be involved in the disease progression of high-risk chronic lymphocytic leukemia (CLL) patients [170]. The oncogenic potential of TRB isoforms in acute leukemias has been recently reviewed and we direct readers to this thoughtful review [171].

TRB1 is over expressed in follicular thyroid carcinoma [172], ovarian cancers [173], and in JAK2V617F mutation-negative patients with essential thrombocytosis [174]. TRB2 expression has also been associated with lung cancers [151], and in melanomas, TRB2 facilitates growth and survival by down-regulating FOXO3a activity [73]. Recent work suggests that TRB2's oncogenic potential is related to its ability to integrate complex signaling pathways including Wnt/*β*-catenin, Hippo/Yes-associated protein (YAP), and C/EBP*α* pathways in liver cancer cells [175]. Thus, tribbles family members play a significant role in cancer development and progression (review in [30]).

## 13. Clinical Correlations

In humans, a gain-of-function TRB3 Glu84Arg (arginine replaces glutamine at position 84) polymorphism is associated with insulin resistance, carotid atherosclerosis, and cardiovascular risk [176–178]. This polymorphism in exon 2 of TRB3 is associated with reduced nitric oxide production in human endothelial cells [179], and plasma levels of C-peptide in humans. TRB3 reduces insulin secretion from pancreatic beta cells and mice over-expressing TRB3 Glu84Arg in beta cells have lower beta cell mass, associated with less proliferation and enhanced apoptosis [68]. The impact of this polymorphism in humans has been recently reviewed [180]. Oberkofler and colleagues evaluated TRB3 levels in the visceral abdominal fat and the liver in obese humans and observed aberrant hepatic TRB3 transcript levels. Additionally, there was a correlation between mRNA levels of TRB3 and plasma insulin [181]. TRB3 is also upregulated in the skeletal muscle of patients with Type II diabetes [54]. Fibrates are ligands of PPAR*α* and commonly used to treat hypertriglyceridemia, and we have previously shown in lymphocytes that fibrates potently augment TRB3 expression, independent of PPAR*α* expression [52]. In both animal and human studies, PPAR*α* ligands are therapeutically efficacious in diabetic nephropathy [182–184]. Fibrates reduce C-reactive protein (CRP) and IL-6 in patients with Rheumatoid Arthritis [185], and we speculate that the beneficial effects of PPAR*α* ligands may be related to augmented lymphocyte TRB3 expression [52, 96].

We have already discussed that variations in TRB1 loci in humans are associated with increased plasma lipoproteins and risk of coronary artery disease [51, 80, 81]. There are also associations of TRB1 gene variants with liver enzyme expression [81], and emerging evidence suggest an association between sleep duration and lipid metabolism. Interestingly, investigators observed that TRB1 gene variants were independently associated with sleep length and lipid metabolism, and there was a significant increase in TRB1 mRNA expression in the peripheral blood mononuclear cells of people restricted to 4 hrs of sleep compared to those with normal sleep duration [186]. Epidemiological studies demonstrate a strong relationship among shortened sleep duration, obesity, and abnormal glucose metabolism [187]. Further work should elucidate whether alterations in TRB isoform expression provide a mechanistic link between sleep duration and metabolic homeostasis.

Autoimmune uveitis is an inflammatory disorder of the eye, and in 2005, Zhang and colleagues discovered in humans that TRB2 is a uveitis-associated autoantigen [188]. Although follow-up studies in uveitis have not been published, TRB2 auto-antibodies have been detected in patients with narcolepsy, a disorder characterized by abnormal daytime sleepiness [189]. Hypocretin (orexin) neurons regulate sleep and wakefulness, and disturbances of the hypocretin system have been directly linked to narcolepsy. Investigators discovered TRB2 auto-antibodies target and lead to the disappearance of hypocretin neurons [190, 191]. Indeed anti-TRB2 antibodies were injected intra-cerebro-ventricularly into mice and they induced narcolepsy-like attacks [192]. Additionally, TRB2 is expressed in tissues of patients with inflammatory bowel disease and may regulate Toll-like receptor 5 signaling [193]. Thus, all TRB family members have been associated with and may modulate diverse human diseases.

The TRB family interacts with and regulates multiple cellular signaling cascades, including the MAPK, insulin signaling-AKT, Notch, cell cycle, and transformation pathways. Our studies suggest that TRB3 may function at the nexus of the ER stress, mTOR, and autophagy pathways. We will first briefly summarize the functions of these pathways and then their interactions with TRB3.

## 14. Endoplasmic Reticulum Stress

The endoplasmic reticulum (ER) folds, modifies, and degrades secretory and transmembrane proteins. Pathophysiological stress conditions, including nutrient deprivation, nutrient excess, altered protein glycosylation, and oxidative stress interfere with normal protein folding. Accumulation of misfolded and unfolded proteins induces their aggregation and subsequent cellular toxicity. Thus, to alleviate the accumulation of ER proteins, a complex intracellular signaling pathway known as the Unfolded Protein Response (UPR) is activated [194–196]. The UPR represses protein synthesis and increases ER chaperone content to restore normal ER function, however when these pathways are overwhelmed by sustained ER stress, the UPR initiates pro-apoptotic pathways [197–199] and autophagy [7].

In mammalian cells, there are three major arms of the UPR: (1) inositol requiring protein-1*α*/X box binding protein-1 (IRE1*α*/XBP-1), (2) protein kinase RNA (PKR)-like ER kinase (PERK), and the (3) activating transcription factor-6 (ATF6) pathways. The PERK pathway rapidly attenuates protein translation, whereas the ATF6 and the IRE1*α*/XBP-1 cascades transcriptionally upregulate ER chaperone genes that promote proper folding and degradation of proteins, allowing the folding machinery of the ER to catch up with the backlog of unfolded proteins. A number of groups, including ours have documented activation of the ER stress response in the diabetic kidney [53, 200] and ER stress is activated in acute kidney injury and other chronic kidney diseases [201–205]. Moreover, over-expression of ER stress-associated molecules, including chemical chaperones that improve ER folding can reduce ER stress and improve outcomes in kidney disease [205–208].

## 15. Mammalian Target of Rapamycin (mTOR)

Another key signaling system in the kidney is mTOR, which is a conserved serine/threonine kinase modulated by growth factors and cellular energy status [9, 209–212]. Mammalian TOR forms two distinct molecular complexes known as mTOR complex 1 (mTORC1) and mTORC2. MTORC1 regulates growth, autophagy, survival and metabolism, whereas the role of mTORC2 in cellular biology is incompletely understood. In podocytes, knockout of mTOR disrupts both mTORC1 and mTORC2 function and alters autophagy flux [213]. Both knockdown and overexpression of mTORC1 in podocytes cause proteinuria in animal models, and human diabetic kidney disease has been associated with enhanced mTORC1 function [214]. Besides regulating the phosphorylation of AKT at Ser^473^ and Thr^450^, mTORC2 also phosphorylates serum/glucocorticoid-regulated kinase-1 (SGK-1) and protein kinase C (PKC) to regulate actin cytoskeletal dynamics, body growth, motility, and survival [215, 216]. Through SGK-1, mTORC2 increases fibronectin expression in high glucose conditions and mTORC2 may regulate nephromegaly, matrix expansion, and excessive sodium reabsorption (potentially involving the epithelial sodium channel ENaC) in diabetic nephropathy [217, 218]. In stress-associated conditions, podocyte-specific knockout of rapamycin-insensitive companion of mTOR (Rictor, a component of mTORC2) causes proteinuria [214]. In podocytes, rapamycin reduces Rictor expression and phosphorylation of AKT at Ser^473^ and this is associated with a reduction in expression of nephrin [219]. Recent work suggests that inhibition of mTORC2 function inhibits inflammation in rodent inflammatory models [220] and reduces IL-6 expression in stem cells [221]. Indeed, many studies have emphasized the importance of tight regulation mTORC pathways in renal pathophysiology.

## 16. Autophagy

Macroautophagy (referred to as autophagy) preserves homeostasis by degrading long-lived proteins and dysfunctional organelles [222, 223]. Autophagy has both cytoprotective or cytocidal effects, and dysregulation of autophagy contributes to podocyte dysfunction in diabetic nephropathy [224]. MTORC1 (which is activated by AKT) inhibits autophagy, and dysregulation of mTORC1 activity disrupts autophagy flux [213]. Autophagy begins with the formation of double-membraned, autophagosomes, which sequester intracellular components. This process is activated by class 3 phosphoinositide-3-kinase and beclin (autophagy-related gene/Atg 6). Cytosolic LC3-I (microtubule-associated protein light chain 3) is conjugated to phosphatidylethanolamine to form LC3-II and recruited to the autophagosomal membrane. Next, autophagosomes fuse with lysosomes to form autophagolysosomes and the intracellular contents, including membrane-bound LC3-II are degraded. Interestingly, fibrates which augment TRB3 expression [52], modify expression of LC3-II [225].

## 17. TRB3 at the Crossroads of ER Stress, mTORC Function, and Autophagy

**Figure 2:**
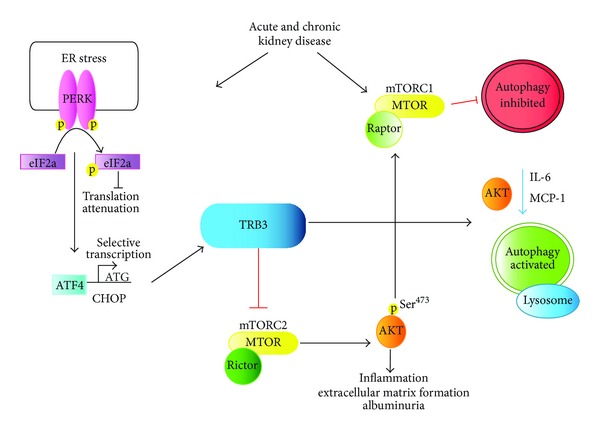
TRB3 at the crossroads of ER stress and mTORC function. Acute and chronic kidney disease are associated with activation of the Endoplasmic Reticulum Stress pathway. During ER stress Protein kinase RNA (PKR)-like ER kinase (PERK) phosphorylates eukaryotic translation initiation factor *α* (eIF2*α*) and inhibits protein translation. Additionally, there is selective translation of activating transcription factor-4 (ATF4), which induces expression of C/EBP homologous protein (CHOP) and drives TRB3 expression. Kidney disease is also associated with activation of mTORC and inhibition of autophagy flux. We propose that in the kidney, TRB3 binds to Rictor and mTORC2 and inhibits phosphorylation of pAKT^Ser473^. This in turn reduces inflammatory gene expression (IL-6 and MCP-1) and potentially modifies activation of mTORC1 and autophagy.

Recent reviews have focused on interactions between ER stress, autophagy, and mTORC, as dysregulation of these pathways can contribute to acute and chronic kidney disease [7, 226]. We have discussed that in renal tubular cells and podocytes that TRB3 preferentially blocks the phosphorylation of AKT at Ser^473^, suggesting that TRB3 may inhibit mTORC2 function. Indeed, our recent unpublished studies suggest that TRB3 may bind to Rictor, to modulate mTORC2 activity. Thus, TRB3 likely functions at the intersection of these complex signaling networks (Figure 2). Autophagy can originate from the ER membrane and be triggered by ER stress [227, 228]. Studies suggest that the IRE1*α*/XBP arm of ER stress activates autophagy, by phosphorylating B-cell lymphoma 2 (Bcl-2) and preventing its interaction with Beclin-1 [229–231]. PERK, another ER stress-associated sensor, mediates conversion of LC3-I (free form) to LC3-II (membrane-bound form), a key step in the induction of autophagy [232]. Additionally, in glioma cells, ER stress induces TRB3, which modifies induction of autophagy [107]. Investigators have speculated that autophagy may supplement ER-associated degradation to reduce the accumulation of misfolded proteins and improve cellular viability [7]. Thus, TRB3 an ER stress-associated protein, by its effects on AKT can modulate mTORC, which in turn activates autophagy. All of these signaling cascades are dysregulated in the injured kidney, and TRB3 is situated at the nexus of these complex pathways. We postulate that manipulation of TRB3 expression is likely to exert significant effects in both acute and chronic kidney disease.

## 18. Conclusions

It is clear that TRB family members exert diverse and somewhat contradictory roles in development, cellular differentiation, survival, metabolic homeostasis, inflammation, and tumorigenesis [233]. TRB isoform knockout mice are viable without obvious developmental or pathophysiological abnormalities; however, when the mice are stressed, the relevance of TRB function becomes more obvious. The diversity of function is quite remarkable and suggests that TRB isoforms may not function in a simple manner by either turning on or off signaling cascades. It is tempting to speculate that TRB family members dampen or augment physiologic signaling cascades that impact cross-talk among diverse tissues including the immune system [234]. This review has emphasized that tribbles homologs interact with pathways that have hundreds of downstream targets; thus, it is likely that they serve as sensors and integrate and fine-tune molecular responses to diverse stimuli. This sensing and integration function may be critical for an organism to survive in an environment of rapidly changing nutrients and inflammatory signals. Once we attain a more precise understanding of TRB function in the kidney and other organ systems, it may be possible to develop novel therapeutics that function similar to TRB homologs that can specifically target and modulate signaling cascades, thereby delicately regulating diverse pathophysiological processes.

## Figures and Tables

**Table 1:** Transcription factors that interact with TRB isoforms.

Transcription factor	Reference
ATF4, ATF5	[66–68]
C/EBP*β*, C/EBP*α*, CHOP	[57, 63, 69–71]
NF-*κ*B	[27, 50]
FOXO1, FOXO3a, FOXP3	[72–74]
PPAR*γ*, RAR*α*, RXR*α*	[47, 64, 75]
